# The impact of artificial intelligence on the person-centred, doctor-patient relationship: some problems and solutions

**DOI:** 10.1186/s12911-023-02162-y

**Published:** 2023-04-20

**Authors:** Aurelia Sauerbrei, Angeliki Kerasidou, Federica Lucivero, Nina Hallowell

**Affiliations:** Ethox Centre, Nuffield Department of Population Health, University of Oxford, Big Data Institute, Old Road Campus, Oxford, OX3 7LF UK

**Keywords:** AI, Doctor-patient relationship, Person-centred care, Empathy, Trust, Shared decision-making

## Abstract

Artificial intelligence (AI) is often cited as a possible solution to current issues faced by healthcare systems. This includes the freeing up of time for doctors and facilitating person-centred doctor-patient relationships. However, given the novelty of artificial intelligence tools, there is very little concrete evidence on their impact on the doctor-patient relationship or on how to ensure that they are implemented in a way which is beneficial for person-centred care.

Given the importance of empathy and compassion in the practice of person-centred care, we conducted a literature review to explore how AI impacts these two values. Besides empathy and compassion, shared decision-making, and trust relationships emerged as key values in the reviewed papers. We identified two concrete ways which can help ensure that the use of AI tools have a positive impact on person-centred doctor-patient relationships. These are (1) using AI tools in an assistive role and (2) adapting medical education. The study suggests that we need to take intentional steps in order to ensure that the deployment of AI tools in healthcare has a positive impact on person-centred doctor-patient relationships. We argue that the proposed solutions are contingent upon clarifying the values underlying future healthcare systems.

## Background

In the Western world, the demand for healthcare professionals is increasing and the population is ageing [1]. As a result, the workload is high, and healthcare systems in developed countries suffer from ever-increasing cost pressures and backlog [1]. The British Medical Association (BMA) warned that COVID-19 further disrupted care pathways in the United Kingdom and that it will take the NHS years to clear backlogs [2]. Against this background, new technologies that can contribute to improved efficiency and ultimately, improved care, are welcome.

Artificial intelligence (AI) tools are increasingly being developed and deployed in the healthcare sector. Technologies which perform as well as, or better than humans already exist [1, 3]. High hopes are placed on AI technology to improve all aspects of healthcare including saving time [3–11]. It is hoped that the time that AI can save can be used to improve doctor-patient relationships and making it more person-centred [12].

Person-centred care is about ensuring that "people's preferences, needs and values guide clinical decisions" [13]. Person-centred care is considered to be the gold standard for doctor-patient relationships [1]. Besides improving satisfaction, decreasing malpractice, and improving employee retention rates, such an approach is said to improve health outcomes [2]. Bauchat et al. argue that empathy forms a cornerstone of person-centred care [14]. They argue that this value is necessary for forming the partnerships and the effective communication that is instrumental to person-centred care [14]. Furthermore, reaching a consensus via shared decision-making without understanding the world from someone else’s standpoint (i.e. empathising with the other) is a difficult task. For these reasons, empathy is foundational to person-centred care, and critical for its practice. [15]. Empathy is also important in so far as it triggers compassion, which can be characterised as “feelings of warmth, concern and care for the other, as well as a strong motivation to improve the other’s wellbeing”(p.875) [16]. As argued by Jeffrey, empathy is a skilled response, whereas compassion is a reactive response [17]. Therefore, empathy is helpful insofar as it acts as a precursor for compassion which allows doctors to act in their patients’ best interest.

Yet, nowadays, time is often insufficient for doctors to develop the type of empathetic and compassionate relationship with their patients that is necessary for person-centred care [18–20]. Artificial intelligence (AI) is commonly cited as a potential solution to the problems faced by healthcare today, including addressing the aforementioned dissatisfaction surrounding the nature of the doctor-patient relationship. AI, is argued, has the potential to “give the gift of time” [21] and could, therefore, allow the doctor and the patient to enter more meaningful discussions with respect to care. For example, tools that enable doctors to outsource certain tasks allow the doctor to spend this saved time on something else, and the hope is that this “will bring a new emphasis on the nurturing of the precious inter-human bond, based on trust, clinical presence, empathy and communication” [12] (p.6).

The idea that the advent of AI in healthcare may help resolve longstanding issues inhibiting the practice of patient-centred care, such as the lack of time, is appealing in theory. However, the impact of the large-scale deployment of AI on the doctor-patient relationship is unclear and difficult to predict [22]. Sceptics have argued that AI may further dehumanise the practice of medicine [23]. AI tools which lack value plurality may encourage a way back to paternalism, only this time, imposed by the AI, rather than the human practitioner [24, 25]. For example, IBM Watson’s role is to rank treatment options based on outcome statistics presented in terms of ‘disease-free survival’ and to show a synthesis of the published evidence relevant to the clinical situation [24]. However, McDougall argues, that this ranking should be driven by individual patient preferences [24]. Others have raised the possibility that the quest for economic efficiency in healthcare will dictate that time saved by the use of AI will be used to push more patients through the system as opposed to enhancing person-centred care [23]. This paper is guided by the following question: how can AI impact the empathetic and compassionate doctor-patient relationship? We identify and critically discuss the main topics in the literature relating to key values relevant to person-centred doctor-patient relationships, with a particular focus on empathetic and compassionate care. Finally, we identify and discuss concrete ways forward proposed in the literature that could support the beneficial deployment of AI in healthcare, and the doctor-patient relationship in particular.

## Methods

### Search strategy and selection criteria

**Fig. 1:**
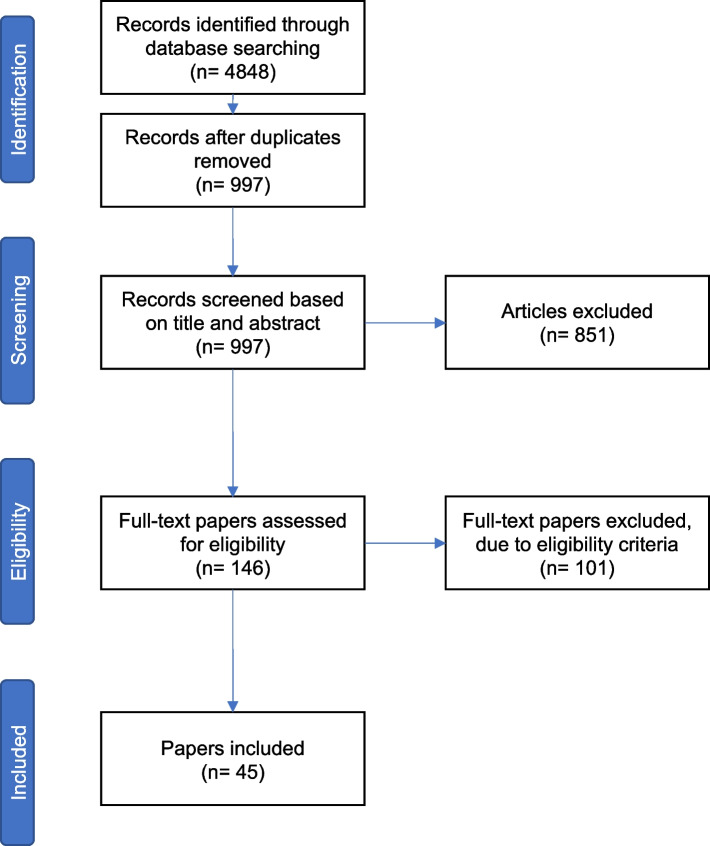
Results of search strategy

We conducted a review of the literature to identify arguments both for the positive or negative impact AI might have on the doctor-patient relationship. Our initial search was conducted using methods commonly associated with systematic reviews in order to ensure a comprehensive coverage of the literature and identify the main topics discussed relating to the impact of AI on the doctor-patient relationship. Searches were conducted in between 1 and 30 April 2021. We included broad search terms to include as many relevant papers as possible (“artificial intelligence”, “machine learning”, “doctor-patient relationship”, “physician–patient relationship”, “therapeutic alliance”, etc.), but included “empathy” and “compassion” as more specific search terms in order to reflect the aim of the research question. We searched 5 differents databases (PubMed, SCOPUS, Web of Science, PhilPapers, Google Scholar). Search results included papers published from database inception to the date of the search. We found 4848 papers. After deleting duplicates, there were 997 papers left. Iterative sessions took place between AS, NH, AK, and FL in order to screen the titles and abstracts and identify the relevant papers. After this initial screening, 146 were identified as potentially relevant. The next step was full-paper screening following which 45 papers were retained. We used an iterative process to synthesise and interpret the data, during fortnightly sessions between all authors. Throughout this process, papers were selected based on the selection criteria discussed with AS, NH, AK, and FL. We decided to select papers written in English, that engaged actively with the question of impact of AI on doctor-patient relationships, and excluded papers that only briefly addressed the question. We deliberately kept the selection criteria broad in order to identify the values emerging from the literature. This enabled us to identify the main issues covered by the literature and identify concrete ways forward to ensure that the use of AI tools benefits the doctor-patient relationship (see Table 1, Fig. 1, and Table 2 for the full search strategy).

**Table 1:** Search

Database	Search terms	Comments	Number of results
PubMed	("ai"[Title/Abstract] OR "artificial intelligence"[Title/Abstract] OR "algorithm*"[Title/Abstract] OR "machine intelligence"[Title/Abstract] OR "machine learning"[Title/Abstract] OR "computer reasoning"[Title/Abstract] OR "computer vision system*"[Title/Abstract]) AND ("doctor patient relation*"[Title/Abstract] OR "physician patient relation*"[Title/Abstract] OR empathy[Title/Abstract] OR compassion[Title/Abstract] OR "therapeutic relation*"[Title/Abstract] OR "therapeutic alliance"[Title/Abstract])	4 searches were conducted using variations of this query (e.g., switching doctor patient into patient doctor)	**910**
PhilPapers	ai OR artificial intelligence OR machine and patient doctor relationship OR empathy OR compassion	Does not allow the same number of search terms as the other databases. 3 searches were conducted using variations of this query (e.g., replacing doctor patient relationship with therapeutic alliance)	**299**
SCOPUS	("ai"[Title/Abstract] OR "artificial intelligence"[Title/Abstract] OR "algorithm*"[Title/Abstract] OR "machine intelligence"[Title/Abstract] OR "machine learning"[Title/Abstract] OR "computer reasoning"[Title/Abstract] OR "computer vision system*"[Title/Abstract]) AND ("doctor patient relation*"[Title/Abstract] OR "physician patient relation*"[Title/Abstract] OR empathy[Title/Abstract] OR compassion[Title/Abstract] OR "therapeutic relation*"[Title/Abstract] OR "therapeutic alliance"[Title/Abstract])	4 searches were conducted using variations of this query (e.g., switching doctor patient into patient doctor)	**1870**
WebofScience	TS = ((ai OR "artificial intelligence" OR algorithm* OR "machine intelligence" OR "machine learning")) AND TS = (("doctor patient relation*" OR "physician patient relation*" OR empathy OR compassion))	Search results were too wide using the same query as PubMed and WebofScience. Narrowed it down to the most relevant/key concepts. 4 searches were conducted using variations of this query (e.g., switching doctor patient into patient doctor)	**1619**
Google Scholar	ai OR artificial intelligence OR machine and patient doctor relationship OR empathy OR compassion	The 150 first (most relevant) results were considered	**150**

**Table 2:** Papers included in the review

Reference	Type of study	Aim of study	Country of origin
(Alrassi 2021) [26]	Scholarly perspective	Presents some of the opportunities and challenges that AI provide. Explains how the role of physicians will evolve in an AI-augmented care environment	USA
(Amann, Blasimme et al. 2020) [27]	Conceptual and ethical analysis	Provides an assessment of the role of explainability in medical AI and ethically analyses what explainability means for the adoption of AI-driven tools into clinical practice	Switzerland, Germany, UK
(Aminololama-Shakeri and López 2019) [8]	Opinion piece	Examines what AI means for breast imaging radiologists and the doctor-patient relationship	USA
(Arnold 2021) [28]	Ethical analysis	Analyses bioethically AI systems and their impact on doctors and patients	Australia
(Banja 2019) [29]	Opinion piece	Advocates for a new field of ethics engaging specifically with health applications, and engages with commonly made bioethical criticism about AI in healthcare	USA
(Bjerring and Busch 2021) [30]	Ethical analysis	Investigates ethically the impact of black-box AI tools on the practice of medicine and patient-centred care	Denmark
(Carter, Rogers et al. 2020) [7]	Research article	Investigates the ethical, legal and social ramifications of using artificial intelligence tools in breast cancer care	Australia
(Chen 2017) [9]	Opinion piece with a case study (University of Hong Kong)	Investigates the role of doctors in future healthcare and the direction medical schools should take to prepare their graduates, in an Asian context	Hong Kong
(Dagher, Shi et al. 2020) [31]	Exploratory review and opinion piece	Assesses the role of wearables in cardiology and outlining the benefits associated with their use	USA
(Davenport and Kalakota 2019) [3]	Research article	Identifies the potential of the use of AI in healthcare and the related potential ethical implications	USA
(Eysenbach, Wright et al. 2018) [32]	Randomised controlled trial (n = 75)	Randomised controlled trial with 75 participants recruited across the United States in order to assess the feasibility and efficacy of using an integrative psychological AI, Tess, to reduce self-identified symptoms of depression and anxiety in college students	USA
(Fogel and Kvedar 2018) [18]	Perspective	Proposes a perspective that AI tools will open the way for a more unified, human experience	USA
(Grote and Berens 2020) [33]	Ethical analysis	Investigates ethically the use of AI tools in clinical decision-making and identification of potential pitfalls of involving machine learning in healthcare	Germany
(Hagendorff and Wezel 2019) [34]	Overview article	Provides a general overview of the current problems that AI and machine learning research and development must deal with	Germany
(Ho 2019) [4]	Opinion piece	Explores the ethical challenges posed by AI tools in healthcare and suggests solutions	?
(Hung, Chen et al. 2021) [35]	Opinion piece	Outlines the benefits of using AI tools in the field of urology	USA
(Johnston 2018) [36]	Commentary	Explores the training needs of future physicians in the age of artificial intelligence	USA
(Jotterand and Bosco 2020) [25]	Commentary	Outlines the conditions necessary for AI to be ethically integrated in healthcare systems	USA
(Karches 2018) [37]	Philosophical analysis	Philosophically analyses why AI tools should not replace human doctors’ judgements	USA
(Kerasidou 2020) [38]	Ethical analysis	Analyses ethically how AI has the potential to fundamentally alter the way in which empathy, compassion and trust are currently regarded and practised in health care	United Kingdom
(Kim, Jones et al. 2019) [39]	Opinion piece	Discusses effects that new technological developments, such as AI, have had on the profession of psychiatry and how teachers can teach trainee psychiatrists the best practices	USA
(Kolanska, Chabbert-Buffet et al. 2021) [40]	Overview article	Summarises AI use in healthcare, its technical, professional, and ethical shortcomings and assesses of how it ought to be used	France
(Kool, Laranjo et al. 2019) [41]	Survey (n = 720)	Conducts a web-based survey of 720 UK GPs' perspectives on whether technology will ever completely replace doctors in providing primary care tasks	United Kingdom
(Lagrew and Jenkins 2015) [42]	Overview and opinion piece	Outlines the future of obstetrics/gynaecology in 2020 including computer-aided diagnoses and proposes a way to thrive in the new system	USA
(Liu, Keane et al. 2018) [43]	Commentary	Outlines how to prepare the future generation of doctors to practice in a health system enabled by artificial intelligence while providing humanity to the machine-patient relationship	United Kingdom
(Luxton 2014) [44]	Review (number of papers used: unspecified)	Identifies and reviews ethical concerns associated with AI care providers (AICPs) in mental health care and other professions. Makes recommendations for the development of ethical codes and the design of AICPs	USA
(Mabillard, Demartines et al. 2021) [45]	Perspective	Discusses the issue of preserving trusting and high-quality relationships between doctors and patients in an era of spread of online information and demands related to accountability placed on healthcare professionals	Belgium, Switzerland
(Manrique de Lara and Peláez-Ballestas 2020) [46]	Narrative review (number of papers used: unspecified)	Provides a narrative review of the bioethical perspectives of big data with a specific focus on the field of Rheumatology	Mexico
(McDougall 2019 [24])	Ethical analysis	Conducts an ethical analysis of the relationship between the ethical ideal of shared decision making and AI systems that generate treatment recommendations	Australia
(Mihai 2019 [47])	Ethical analysis	Investigates ethically which, if any, aspects of medicine—currently or in the future—can and ought to be left in the hands of AI	USA
(Molnár-Gábor 2020) [48]	Research article	Examines the practical and ethical issues that the application of AI raises for people and society	Germany
(Nelson, Pérez-Chada et al. 2020) [10]	Qualitative study using semi-structured interviews (n = 48)	Investigates how patients view the usage of AI for skin cancer detection and how they conceptualise the technology. Qualitative study with semi structured interviews conducted in hospitals in Boston, USA. 48 patients were enrolled	USA
(Niel and Bastard 2019) [22]	Perspective	Provides an overview of evidence on medical artificial intelligence relevant to the field of nephrology. Defines core concepts, recent clinical applications and provides a perspective on future considerations including ethical issues arising	France
(Printz 2017) [49]	Commentary	Summarises the evidence of the time saving capabilities of Watson for Oncology and provides a perspective on AI as an assistant for treatment decisions	USA
(Rainey and Erden 2020) [50]	Ethical analysis	Identifies issues with the application of neural technologies in psychiatry and urges caution, especially regarding normative issues	UK
(Sparrow and Hatherley 2020) [23]	Opinion piece	Provides a critical analysis of the positive discourse surrounding the doctor-patient relationship and implementation of AI in healthcare	Australia
(Szalai 2020) [51]	Research article	Explores theoretically and practically the possibility of AI-based addendum therapy for borderline personality disorder and identifies its potential advantages and limitations	Hungary
(Trachsel, Gaab et al.) [52]	Research article	Explores the use of chatbots and AI tools as supplements to psychotherapy delivered by humans, and as supervised primary treatments. Discusses how ethical guidelines and standards for AI in mental health are relevant in the ethics of AI in psychotherapy	Switzerland, USA
(Triberti, Durosini et al. 2020) [53]	Perspective	Explores the “third wheel” effect that AI introduces in healthcare and identifies the impact of this effect created by AI on the healthcare process, with a focus on future medical practice	Italy
(Tripti and Lalitbhushan 2020) [54]	Opinion piece	Explores the future role of medical doctors in an AI-augmented environment and the related implications on medical education	India
(Wartman 2019) [55]	Commentary	Identifies challenges facing medical education and ways forward to address these challenges, including the preservation of the doctor-patient relationship in an AI-augmented world	USA
(Wartman 2019) [56]	Opinion piece	Explores ethically how medical education systems should adapt to the integration of AI systems in healthcare	USA
(Young, Amara et al. 2021) [57]	Mixed-method systematic review (n = 23)	Explores patient and general public attitudes towards clinical artificial intelligence using a mixed-method systematic review in biomedical and computational databases. 23 papers met the inclusion criteria	USA
(Yun, Lee et al. 2021) [58]	Behavioural study (n = 350) and neural study (n = 22)	Explores (1) Behavioural and (2) neural consumer responses to human doctors and medical artificial intelligence. Study (1) recruited 350 Amazon Mechanical Turk (MTurk) and study (2) recruited 22 participants in their twenties	Korea
(Žaliauskaitė 2020) [59]	Theoretical analysis	Discusses challenging aspects of patients’ right to autonomy in the context of technologies and innovation and the role of the implementation of legal instruments against this background	Lithuania

The aim of this paper was to be “evidence-informed” rather than “evidence-based”, meaning that evidence is understood as “contextually bound but also individually interpreted and particularised within that context” [60]. Therefore, we took a critical approach to reviewing the literature [61]. We chose this approach based on the premise that the question we are asking requires “clarification and insight” as opposed to “data”, in which case a systematic review would have been more appropriate [61]. To this end, as explained above, the approach we adopted was an interpretive and discursive synthesis of existing literature based upon purposive selection of the evidence [61].

## Results

### How are decision made in doctor-patient relationships?

Patient involvement in decision-making is a central aspect of person-centred care [24]. Increasing the patient’s autonomy by encouraging their involvement in decision-making processes is a powerful pushback against the outdated paternalistic model of care [62]. Elwyn et al. argue that shared decision-making rests on the acceptance that individual self-determination is a good, and therefore desirable goal [63]. Thus, supporting patient autonomy is important within this framework [63]. 

Some AI tools may have the potential to increase patient autonomy, and therefore the practice of shared decision-making [59]. Zaliauskaite discusses patient autonomy within the context of technological advances and argues that an effective way to ensure patient’s autonomy is the implementation of legal instruments such as informed consent, advance directives and Ulysses contracts (a contract to bind oneself in the future) [59]. She suggests that technologies such as mobile apps that are used by patients for self-monitoring (collecting any form of health data) may increase autonomy and, in the best case scenario, shift the doctor-patient relationship towards a customer-service type format, where both sides have a balanced distribution of rights and responsibilities, and thereby an equal input/share in the decision-making process [59]. However, one could argue that it is questionable whether a balanced distribution of rights and responsibilities is feasible in a doctor-patient relationship which is commonly characterised by the vulnerability of the patient towards the doctor and epistemic imbalances. Additionally, there seems to be a risk that such a relationship becomes purely transactional and subject to market pressures. In contrast, De lara et al. present bioethical perspectives within the context of big data and data processing in rheumatology and argue that relationships must preserve fiduciary duties, which implies a power imbalance. According to them, this is necessary in order to protect the promise of an ethical relationship of trust between doctors and patients [46]. 

A more fundamental problem arises when considering the type of patient autonomy an AI tool can support within a framework of shared decision-making. It is unclear how an algorithm could take preferences of different people (e.g. regarding treatment goals) into account [24]. This could give rise to a new form of paternalism in which the AI makes decisions on behalf of patients and doctors. The difference with the old form of paternalism is that this time, the paternalistic relationship would be vis-à-vis the AI, not the doctor. In other words, “doctor knows best – but the computer knows more and makes fewer mistakes” [24, 28]. This new form of paternalism would be fundamentally at odds with the principle of shared decision-making. Jotterand et al. (2020) as well as Rainey and Erden (2020) share similar concerns, explaining that in the context of neurotechnology in psychiatry, AI tools are potentially dangerously reductive. This is because they are unable to comprehend social, psychological, biological, and spiritual dimensions. Therefore, they too, argue that AI tools should be designed to allow for value plurality [25, 50]. 

McDougall uses IBM Watson as an example to argue that AI machines should be designed and built in a way that allows for value plurality, namely the ability to take into account different patients’ preferences and priorities. IBM Watson’s role is to rank treatment options based on outcome statistics presented in terms of ‘disease-free survival’ and to show a synthesis of the published evidence relevant to the clinical situation [24]. However, McDougall argues, that this ranking should be driven by individual patient preferences [24] (e.g., one patient might choose further treatment whereas another might choose palliation). Without taking into account value plurality, there is a real risk of the AI’s decisions undermining the patient’s autonomy [24]. 

Black box AI tools are arguably particularly threatening to shared decision making as the absence of explainability might hurt patient autonomy by preventing the patient from making informed decisions [48]. Doctors may have more time to spend talking to patients, but if they are unable to provide the necessary explanations about certain treatment decisions/ prognoses and/or diagnoses suggested by the AI, the benefits of extra time may be limited [7]. 

In summary, the emerging literature is divided on whether AI will enhance the doctor-patient relationship by encouraging shared decision-making through increased patient autonomy or create a new form of paternalism by hindering value-plurality. The next section will focus on the impact of AI on another important aspect of person-centred care, the practice of empathetic care and how it relates to efficiency.

### The tension between empathetic and efficient doctor-patient relationships

Bauchat et al. argue that empathy forms the cornerstone of person-centred care. Multiple studies support these claims [64–67]. Empathy can be described as “…the ability to understand a person’s standpoint, their experience of illness and, through this cognitive resonance, feel motivated to help them…”(p.1) [68]. Empathy facilitates doctors’ understanding of the disease from the standpoint of the patient, as well as individual patients’ values and goals [15]. However, doctors and patients must be able to enter meaningful discussions in order for doctors’ to be able to appreciate and comprehend the patient’s standpoint. The practice of empathy therefore requires time [26, 69]. 

The medical literature is rich in accounts promoting AI as a great time saver creating space for more meaningful and empathetic relationships to be developed with patients [3, 8–10, 18, 31, 35, 70]. There is already some evidence to suggest that AI can save doctors’ time. Printz explains that the AI tool Watson for Oncology needs 40 s to capture and analyse data, then generate treatment recommendations based on the available data [49]. In comparison, manually collecting and analysing the data takes on average 20 min, decreasing to 12 min when oncologists become more familiar with cases [49]. It is unclear, however, if this saved time will be used to enhance the doctor-patient relationship.

In his book “Deep Medicine”, Topol argues that AI tools have the potential to help doctors in a wide array of tasks and therefore could free up time which could be used to build a positive relationship with the patient [19]*.* Aminololama-Shakeri and Lopez [8] argue that AI is the next step towards a more patient-centred system of care in breast-imaging. They observe that because radiologists will have more time to spend with their patients, this will enable them to prioritise the relational aspects of their work. This newfound time, they argue, will also enable radiologists to focus on treatment on top of diagnosis. They explain that this could be achieved by creating a form of hybrid training which would incorporate imaging to medical and surgical oncology training, which has already been suggested for cardiovascular surgeons [8]. This account seems somewhat paradoxical, as if time saved using AI tools results in radiologists taking on other tasks such as treatment, it is unclear how this, in itself, improves the empathetic doctor-patient relationship.

Sparrow and Hatherley [23], in contrast, suggest that the economics of healthcare, especially in for-profit environments but also in the public sector, will dictate that more patients will pass through the system and more tasks will need to be taken on by individuals. They argue that there is no reason to believe that the time saved by the use of AI will result in more empathetic doctor-patient relationships but rather it will allow higher patient throughput. Topol is certainly not oblivious to market laws and has suggested that doctors must get together to create a movement demanding that time saved is not used to squeeze more patients through the system [71]. Sparrow and Hatherley have a pessimistic outlook on the ability of doctors to initiate change, at least in the US context. Using several historical examples (such as universal basic healthcare), they argue that doctors have been unable to motivate any changes under any administration in the US [23]. 

Whether time saved will be used to promote empathetic relationships or used to increase throughput of patients will largely depend on how much value healthcare systems will place on empathy as a healthcare value versus efficiency. This is to a large extent an empirical question, and therefore, more research is needed to determine this. Of course, achieving patient-centred empathetic care is also dependent upon patients being able to trust their doctors and their recommendations. The next section addresses the impact of AI tools on the doctor-patient trust relationship.

### The role of explainability and its impact on the doctor-patient relationship

AI tools can be seen as a new, third actor, in the two-way doctor-patient relationship. Just as the doctor-patient relationship is founded on trust [72], patients and doctors alike must be able to develop a trust relationship with the AI tool they are using. In order for someone to warrant trust, they need to demonstrate their trustworthiness. One way of doing this is by indicating their reliability. In the case of AI this might require features such as explainability, validity and freedom from algorithmic bias, as well as clear pathways of accountability [73]. AI tools do not always conform to these values. For example, AI tools are not necessarily built to be transparent [30, 34]. The continuous search for increased accuracy often compromises AI’s explainability**.** The best AI tools, from a performance perspective are, therefore, not necessarily transparent [27]. Triberti et al. argue that the lack of explainability could lead to a phenomenon of “decision paralysis” due to the trust issues for the users of the AI tool, generated from the lack of explainability [53]. 

The issue of AI explainability raises a number of ethical questions including, whether it would be justifiable to dismiss the use of highly efficient AI on explainability grounds. Ho argues that uncritical deference to doctors over (unexplainable) AI tools that have outperformed humans may lead to preventable morbidity and is ethically irresponsible [4]. According to this view, the deployment of an AI tool might end up becoming compulsory as a matter of due diligence [40] and its use might effectively become an epistemic obligation [30].

Others argue that explainable AIs might give rise to a more productive doctor-patient relationship by increasing the transparency of decision-making. Mabillard et al. [45] propose a framework of “reasoned transparency” which entails elements such as abundant communication about AI tools and services and reassurance on data confidentiality. In a reasoned transparency framework, explainable AI is seen as a powerful tool due to its increased transparency, and therefore, its ability to generate trust relationships, between the AI the doctor and the patient. This is because the doctor can give much more precise information and explain, for example, which specific parameter played a role in an AI tool’s prediction [45]. 

Even in cases where AI tools’ output is not directly explainable, probabilities are and doctors may be able to justify diagnoses and explain procedures in a manner understandable to patients, even if the latter are unfamiliar with statistical jargon. Similarly, patients might be happy to develop a trust relationship with the AI tools that they use as part of their self-management and retain a trust relationship with their doctor, on the grounds of explanations of probabilities and statistics the doctor provides. This will be dependent on medical education changing accordingly, as will be discussed below.

Kerasidou suggests that in an AI-assisted healthcare system, there might be a shift away from human-specific skills if patients and healthcare systems start to value the increased accuracy and efficacy of AI tools over relational values such as interpersonal trust [74]. In this context, one could argue that AI tools do not necessarily need to be explainable (or transparent) to improve the doctor-patient relationship, especially if they systematically out-perform human doctors. Patients and doctors alike might start perceiving trustworthiness as based on the level of certainty or accuracy offered by AI tools, as opposed to a high level of transparency. According to Banja, if our main interest is the accuracy of clinical decision making, then “just like Watson on *Jeopardy!*, AI is going to win the machine-versus-human contest every time” [29] (p.34). He further suggests that AI technologies are held to an unfairly high standard as excessive attention is paid to their errors as opposed to human errors. In this context, one could argue that AI tools do not necessarily need to be explainable (and therefore transparent) to improve the doctor-patient relationship, especially if they systematically out-perform human doctors. Patients and doctors alike might start basing their trustworthy relationship on the understanding that AI tools offer a high level of certainty, as opposed to a high level of transparency. De Lara et al. explains that medicine is already full of black boxes [46]. For example, not all doctors and patients need to understand how electromagnetic radiation works when dealing with an MRI machine. Bjerring and Busch, however, argue that AI is a different type of black box [30]. They explain that, currently, there is always a human in the loop who is able to give an explanation of how technology works (for example, there will be an engineer able to explain how an MRI machine works), but this cannot be said of some AI systems [30]. 

Beyond issues relating to accuracy and efficiency, explainability is also linked with the problem of accountability. Carter et al., discussing AI-assisted breast cancer diagnostic tools, suggest that a lack of explainability is problematic if the doctor is expected to take responsibility, i.e., be accountable, for decisions involving AI systems [7]. Furthermore, it is unclear to whom responsibility for AI-mediated decisions should be delegated, and how the interactions between AI tools and doctors will develop given this uncertainty [33]. A shift in the attribution of responsibility from the doctor to other stakeholders (e.g. AI developers, vendors) may have a negative impact on the doctor-patient relationship as traditional systems of accountability become compromised.

Generally, therefore, the argument is that due to their lack of transparency and difficulties surrounding systems of accountability unexplainable, black box AI could have a negative impact on the doctor-patient relationship. On the other hand, the use of highly efficient, albeit unexplainable, AI tools could be morally justified – and indeed encouraged – given the potential health benefits resulting from their accuracy. Further research is necessary to determine how different types of AI tools should be used in different clinical situations.

So far, we have outlined the main debates in the literature regarding the likely impact of AI tools on the practice of person-centred, doctor-patient relationships. The following sections present suggestions found in the literature which aim to ensure that the implementation of AI tools benefits the doctor-patient relationship.

### Solutions

The literature suggests that (1) ensuring that AI systems retain an assistive role in clinical encounters and (2) adapting medical education to ensure future doctors are prepared for an AI-assisted work environment may improve doctor-patient relationships.

#### What is the role of AI tools in healthcare?

Many have observed that the impact of AI on person-centred care is likely to depend on the role it occupies in clinical contexts; assisting versus replacing human practitioners. The ideal role for AI in healthcare is currently unclear [53]. Yun, Lee, et al. shed some light on current dynamics between AI machines and people [58]. Using a combination of a behavioural and MRI-based neural investigation, they found that, generally, participants demonstrated an intention to follow the advice of a human doctor rather than an AI machine. In the behavioural experiment, they found that participants' self-reported willingness to follow AI recommendations increased if the AI was able to conduct personalised conversations, but they were still more likely to state they preferred human doctors’ recommendations. In a second experiment using neuroimaging, they identified the neurocognitive mechanisms that underlie responses to personalised conversation conducted by AI tools versus human doctors [58]. They found inconsistencies with the first experiment: participants’ brain responses showed apathy towards medical AI tools, even when using personalised conversational styles. Human doctors, in contrast, elicited a pro-social response. This experiment suggests a future where AI may be better accepted by patients if it acts as an assistant to human doctors rather than replaces them. Furthermore, a review investigating patients’ and publics’ attitudes towards AI found that while AI was viewed positively overall, participants strongly preferred AI tools to be assistive, with only a minority believing that the technology should either fully replace the doctor or not be used at all [57]. 

Several studies in the field of mental health support the view that AI can only have a positive impact on the doctor-patient relationship in an assistive role by improving openness, communication, and avoiding potential complications in interpersonal relationships [32, 51, 52]. For example, supporting the view that AI can only positively impact the doctor-patient relationship in an assistive role, Szalai argues that AI-based addendum therapy for patients with borderline personality disorder can be beneficial [51]. This is done using algorithms capable of identifying emotional tone of a narrative and fine-grained emotions. Patients may be more willing to disclose information to the AI than to the human doctor, even when they know that the human doctor can access the information. On the other hand, Luxton warns of the risk of AI tools replacing human doctors arguing that the imperfection of the psychotherapist is an essential part of the healing process. He argues that patients must be warned, and stakeholders must be mindful of the ethical implications of the use of these types of AI tools for mental healthcare [44]. 

There is some evidence that clinicians also believe that assistive AI may have a positive role to play in doctor-patient relationships. An exploratory survey conducted with general practitioners in the UK showed that they too believe in a restricted role of AI within general practice [41]. Opinions were extremely varied as to how AI tools may be incorporated in practice. The overwhelming majority of the respondents were sceptic as to the ability of AI tools to help with diagnoses, save time, etc [41]. Interestingly, however, the study shows that the views of GPs are often far removed from those of AI experts [41]. The latter forecast that primary care will be radically transformed as evidence suggests that mHealth tools enable patients to monitor key variables without the need for traditional check-ups. Mihai warns, though, that this may backfire as patients might worry obsessively about continuous monitoring which is likely to be counterproductive [47], presumably because this might unnecessarily increase the demand on healthcare services as a result. To mitigate this phenomenon, strategies could be put into place where the readings are automatically sent to the doctor but only visible to the patient if they so wish, and alerts are only sent in cases of emergency. These views suggest that AI tools can only have a positive impact on the doctor-patient relationship if they are used in an assistive manner, that is, ensuring human-to-human empathetic relationships are preserved. Karches argues that AI should not replace the human doctor, particularly in caring for people with chronic and terminal illnesses, as human doctors are able to “offer wisdom and compassion from his or her own experience of being human” [37] (p.108). Therefore, (preferences for) the use of AI may be influenced by illness type [37] and level of empathy required [43]. 

In summary, if public acceptability of AI tools is a concern, current evidence seems to suggest that introducing them in an assistive capacity in healthcare is less likely to have a negative impact on the doctor-patient relationship. Assistive tools, especially explainable ones, may even support empathetic and trust-based doctor-patient relationships by giving sufficient space to the doctor to perform their role. They can also promote shared decision-making by allowing doctors and patients to take their own preferences into account. It is likely that the use of AI tools in healthcare may spread as patients and doctors adapt to their use, indeed Banja observes that humans are robust anthropomorphisers and thus the acceptance of AI tools is very likely to increase with time [29]. 

#### What are medical professionals’ educational needs in an AI-based system?

Whether AI impacts the doctor-patient relationship positively or negatively depends on the structural aspects of the healthcare system within which AI is being deployed. For example, in order for AI to help promote empathetic doctor-patient relationships, it needs to be deployed within a system that already supports empathy as a core healthcare value [38]. This arguably starts with defining appropriate medical curricula. Tripti and Lalitbhushan suggest that it is important that doctors learn how to interact with AI systems and large data sets while at the same time providing humane and compassionate care [54]. These relational skills will define their role in future healthcare given that AI systems are likely to take over some of the knowledge aspects of their job [43, 54]. In other words, they argue that AI tools are likely to cognitively surpass humans, making it necessary for human providers to adapt to working together with AI tools [54]. 

Kolanska et al. go further by arguing that the doctor’s role should evolve to be closer to an engineer, that is, with an understanding of big data and computer science [40]. In the context of psychiatry, Kim et al. explain that most medical schools have lagged behind shifts brought about by the increasing use of technology. Given that AI is likely to assist psychiatrists, Kim et al. argue that medical education ought to reflect this newly defined role for both doctors and AI in the provision of healthcare [39].

Another approach to preserving the doctor-patient relationship in the age of AI is to increase the focus on soft skills in the medical curriculum [18, 36]. Besides the importance of AI literacy, Wartman et al. also suggest that empathy and compassion are skills that should be cultivated or taught throughout the curriculum and actively kept at the centre of medical practice [55, 56]. Lagrew and Jenkins explain that besides the importance of the study of new technologies, the best doctors will be those who understand how it “feels” to be a patient [42]. Chen suggests a related approach, she observes that technical knowledge and skills are no longer the exclusive domain of the medical profession, as knowledge is now easily accessible to the public and AI is developing diagnostic skills. Thus, she argues, other relevant competencies should be further developed, such as the ability to know when and how to apply knowledge in order to best help the patient in a compassionate manner [9]. Alrassi et al. similarly underline the importance of selecting medical students who have high empathy, communication skills, and emotional intelligence [26] in order to ensure appropriate care in a future relying increasingly on emotionless AI tools.

In summary, adapting medical education appropriately is seen as crucial to ensure that empathetic care, trust relationships, and shared decision-making are preserved in AI-assisted healthcare systems. It is argued that this can be achieved through an increased focus on data science in the curriculum whilst preserving a strong emphasis on relational skills.

## Conclusions

The literature shows that AI has the potential to disrupt person-centred doctor-patient relationships. AI tools could support the practice of shared decision-making by increasing patient autonomy. Alternatively, AI tools could harm shared decision-making by creating a new form of paternalism due to their lack of value plurality. Similarly, AI tools have the potential to improve the practice of empathetic care by saving time. However, it is unclear if the saved time will be used to practice empathetic care or used for other activities including pushing more patients through the system. Trustworthy relationships could also be affected by the use of AI tools. Generally, explainable AI tools are considered to be valuable tools for supporting trust relationships given their transparent nature. Blackbox AI tools, however, could negatively impact trust relationships due to their inherent complexity.

The literature proposes several ways forward to ensure that AI tools support, rather than hinder, person-centred doctor-patient relationships. A handful of studies suggest that when AI is used as an assistive tool, this may have a positive impact on the doctor-patient relationship (e.g. Eysenbach et al.; Szalai). However, it is argued that patients and doctors may be unlikely to accept a shift to AI-led medical care, and such a shift could harm the doctor-patient relationship as AI tools are incapable of reproducing inherently human qualities of empathy and compassion. In the longer term, the debate is still open with regards to how human preferences for AI-led healthcare will evolve. Patients and doctor alike might start favouring the increased accuracy of AI-led care. However, current evidence regarding human preference points to the fact that this is not yet the case. There is broad agreement in the literature that the impact of AI on the doctor-patient relationship will influence and be influenced by the education of medical professionals. Most authors seem to suggest that medical education should focus on AI literacy and emotional intelligence, with some emphasising the importance of one over the other. This combination underlines the importance of upholding empathetic care while ensuring that patients understand the tools used by the doctor, therefore contributing to the development of trust relationships.

Prior to concluding we should note that this paper has some limitations. It focuses on academic literature published in English, therefore, although it aimed to be comprehensive, it is possible that some issues have been overlooked. Second, we have not focussed on a specific type of AI tool. It is possible that relevant issues may vary and depend on the specific usage and role of the tool. Furthermore, most of the debates surrounding the use of AI in healthcare are speculative given the current limited adoption of AI tools. While there are some implementation studies available, few focus specifically on the doctor-patient relationship, and only those would have been selected for this literature review, given our search terms. Finally, there was no patient and public involvement (PPI) as part of this project. We encourage researchers undertaking future studies on this topic to involve patients.

It is clear that AI could act as a disruptor to healthcare systems, it is therefore necessary to think about its exact place and role within wider healthcare systems to ensure that its deployment is beneficial for the doctor-patient relationship. On this basis we argue that healthcare systems and related stakeholders, including citizens and policy makers, need to consider the type of values they wish to promote in an AI-augmented healthcare system, and workflows should be adapted accordingly.

## Data Availability

Not applicable.

## Abbreviation

AI: Artificial Intelligence
